# Latent profile analysis of moral resilience among clinical nurses and its association with ethical behavior: a multi-hospital cross-sectional study in China

**DOI:** 10.3389/fpubh.2026.1787493

**Published:** 2026-06-04

**Authors:** Xiaohan Shi, Ying Zhang, Hongjuan Lang, Rui Wang, Yang Wang, Aiping Sun, Yuan Hui

**Affiliations:** 1 Healthcare Center for Military Personnel, Tangdu Hospital, The Fourth Military Medical University, Xi’an, Shaanxi, China; 2 Department of Nursing, The Fourth Military Medical University, Xi’an, Shaanxi, China; 3 Department of Ophthalmology, Tangdu Hospital, The Fourth Military Medical University, Xi’an, Shaanxi, China

**Keywords:** clinical nurses, ethical behavior, influencing factors, latent profile analysis, moral resilience, relevance

## Abstract

**Objective:**

To explore the categories of the moral resilience latent profile of clinical nurses, analyze the influencing factors of different categories, and explore the correlation between them and ethical behavior.

**Methods:**

By convenience sampling, nurses from 16 level-two-and-above hospitals in China were selected as the research subjects from May to June 2025. The Chinese version of the moral resilience Scale and the revised version of the Nurses’ Ethical Behavior Scale were used to survey the enrolled research subjects. Latent Profile Analysis (LPA) was performed in Mplus 8.3 to identify subgroups based on moral resilience scores. Model selection relied on the lowest AIC, BIC, and aBIC values, entropy > 0.80, and significant LMR-LRT/BLRT statistics.

**Results:**

The scores of moral resilience and ethical behavior of clinical nurses were (44.27 ± 5.33) and (41.08 ± 6.34) respectively. The clinical nursing ethics elastic latent section is divided into three categories: Category 1 (C1) is the “high moral resilience group,” accounting for 28.1% (95 cases); Category 2 (C2) is the “medium moral resilience group,” accounting for 46.2% (156 cases); Category 3 (C3) is the “low moral resilience group,” accounting for 25.7% (87 cases). Univariate analysis showed that there were significant differences in nursing age (χ^2^ = 12.916, *p* = 0.044), hospital level (χ^2^ = 14.006, *p* = 0.007), whether they were specialized nurses (χ^2^ = 16.721, *p* < 0.001), and whether they participated in ethical training (χ^2^ = 14.237, *p* < 0.001) among the three categories of nurses. Logistic regression analysis showed that nursing age, hospital level, whether they were specialist nurses, and participation in ethics training were the influencing factors of moral resilience (*p* < 0.05). The results of pairwise comparison showed that the level of ethical behavior of clinical nurses in group C1 was significantly higher than that in group C2 (*p* < 0.01) and that in group C3 (*p* < 0.01), and the level of ethical behavior of clinical nurses in group C2 was significantly higher than that in group C3 (*p* < 0.01).

**Conclusion:**

Latent profile analysis was applied in this study. The moral resilience of clinical nurses was divided into three categories. The moral resilience of different profiles is closely related to the level of ethical behavior of clinical nurses. Based on these findings, healthcare institutions should implement targeted intervention strategies tailored to the heterogeneous characteristics of nurses’ moral resilience, enhancing their moral resilience levels by optimizing organizational support and ethics training mechanisms; this approach subsequently standardizes clinical ethical behavior and improves patient care quality, offering significant implications for strengthening human resource management in health systems and advancing public health practice.

## Introduction

The concept of moral resilience, systematically articulated by Rushton et al., refers to the capacity of healthcare professionals to effectively navigate, adapt to, and recover from ethical dilemmas. It serves as a crucial psychological resource that enables nursing professionals to uphold moral integrity and adhere to professional values in complex clinical contexts (1, 2).

In the context of the rapidly developing medical system in our country, the nursing group faces multiple ethical challenges, such as an unbalanced nurse—patient ratio, an extraordinary workload, and frequent career risks (3, 4). Nearly 90% of nurses suffer from occupational exposure, tense doctor—patient relationships, and other dilemmas. These factors together constitute a severe test of nurses’ moral resilience (5–7).

Ethical behavior refers to actions that conform to moral norms and have moral evaluation significance under the control of certain moral consciousness. It is the external and concrete expression of moral cognition and moral emotion. Moral resilience, as the ability of individuals to effectively cope with, adapt to, and recover from ethical dilemmas, is one of the important psychological factors affecting the level of clinical nurses’ ethical behavior (8, 9).

In recent years, within the context of ethical behavior, Chinese nursing academia has begun to deeply explore the concept of moral resilience, aiming to uncover the internal mechanisms by which nurses resist ethical dilemmas while fulfilling their ethical responsibilities. Key research findings indicate that moral resilience is not only a significant driver of ethical behavior but also shows a significant positive correlation with it. Nurses with high moral resilience are more inclined to adhere to ethical principles when facing resource constraints or value conflicts, demonstrating higher quality in ethical decision-making and greater consistency in behavior, thereby effectively buffering the negative impact of ethical dilemmas on ethical conduct (10). The underlying mechanism may lie in the fact that moral resilience enhances an individual’s moral efficacy—the belief in one’s ability to handle ethical issues—and moral sensitivity, thereby increasing the likelihood and capacity to make judgments and take actions that align with ethical standards in complex situations. Conversely, positive feedback from ethical behavior, such as gratitude from patients and recognition from the team, can reinforce nurses’ moral identity, further enhancing their moral resilience and creating a virtuous cycle.

Clinical surveys show that the average score of moral resilience of nurses in China is only 40.73, which is at a medium level and is significantly negatively correlated with moral distress (11). This indicates that nurses still face great pressure when dealing with ethical conflicts. For individual nurses, high moral resilience can help reduce job burnout, improve job satisfaction, and avoid moral fatigue and psychological exhaustion caused by persistent ethical dilemmas (12). Similarly, nurses with strong moral resilience are more sensitive in identifying ethical issues and making ethical decisions, thereby protecting patients’ rights and improving the quality of nursing (13, 14). Therefore, conducting research on moral resilience and exploring effective intervention strategies to enhance nurses’ moral resilience are of great significance for improving nurses’ professional wellbeing, ensuring patients’ safety, and optimizing the quality of medical services.

Previous studies focusing on Chinese nursing populations have predominantly employed variable-centered approaches, which, while yielding mean scores of moral resilience (11, 15), fail to capture inter-subgroup variations and consequently limit the development of targeted intervention strategies. The majority of existing research utilizes variable-centered methods to analyze moral resilience, which not only makes it difficult to reveal heterogeneous characteristics within the population but also lacks identification of latent classes of nurses’ moral resilience. Latent Profile Analysis (LPA), an individual-centered methodological approach, enables the classification of study populations into distinct latent profiles based on multiple indicators, clarifies the characteristics of different sub-groups, and provides a theoretical basis for formulating stratified intervention strategies.

According to Conservation of Resources Theory, individuals strive to acquire, maintain, and protect their valuable resources. Nurses’ resource reserves and allocation strategies across various dimensions of moral resilience (cognition, emotion, behavior, resources) differ, theoretically forming distinct resource combination patterns, i.e., different latent profiles. From the perspective of Conservation of Resources Theory, ethical behavior consumes psychological resources. A high and balanced reserve of resilience resources, or a prominent reserve of key resources (such as cognitive appraisal and behavioral persistence), can more effectively support sustained and stable investment in ethical behavior, preventing moral withdrawal caused by resource depletion (16). However, the acquisition and allocation of these individual resources do not occur in isolation but are shaped and developed through the dynamic interplay between the individual and the environment within specific social contexts. Social Cognitive Theory emphasizes the interaction between the individual and the environment. The development of personal resilience is influenced by experience (17), while a supportive organizational ethical climate serves as a key environmental resource that may foster the formation of more optimal resilience profiles (18).

Together, COR Theory and Social Cognitive Theory provide a complementary framework: the former accounts for individuals’ motivation to preserve psychological resources in the face of ethical demands, while the latter explains how professional experience and the organizational environment shape the development of those resources over time.

Based on the above discussion, this study proposes the following specific hypotheses:

*H1* (Profile Existence Hypothesis): Clinical nurses’ moral resilience comprises multiple latent profiles with significant differences.

*H2* (Profile Differentiation Hypothesis): Nurses belonging to different moral resilience profiles demonstrate significant differences in demographic characteristics (e.g., age, years of nursing experience, department) and work environment characteristics (e.g., ethical climate, work stress).

*H3* (Behavioral Prediction Hypothesis): Nurses belonging to different moral resilience profiles exhibit significant differences in their level of ethical behavior. Nurses with a “high-balanced” or a “cognition-behavior-dominant” resilience profile are expected to demonstrate the highest level of ethical behavior.

Therefore, this study aims to employ latent profile analysis to investigate the moral resilience of clinical nurses across 16 hospitals in China. Specifically, it seeks to identify distinct latent profiles, determine the influencing factors associated with each class, and elucidate the relationship between these profiles and ethical behavior. The findings are intended to provide novel insights and a theoretical basis for developing targeted intervention strategies to enhance the level of ethical behavior among clinical nurses.

## Research subjects and methods

### Study subjects

This study adopted a cross—sectional survey design using a stratified convenience sampling method. First, according to China’s administrative divisions and the distribution of medical resources, eight provinces spanning diverse geographical regions, including East China, North China, and Western China, were selected. This was done to ensure the sample’s representativeness in terms of regional socioeconomic levels and healthcare environments. Second, within these provinces, 16 hospitals (including tertiary and secondary hospitals) were conveniently chosen as study sites based on the research team’s established collaborative networks and the feasibility of on—site data collection. Finally, clinical nurses were conveniently recruited from these hospitals with the assistance of the nursing departments.

Inclusion Criteria:

1. Registered nurses with more than one—year full—time clinical work experience;
2. Age ≥ 18 years old;
3. Good physical and mental health with no previous history of mental illness;
4. Giving informed consent and voluntarily participating in this investigation.

Exclusion Criteria:

1. Refresher nurses, standardized training nurses, assistant nurses, and nursing practice students;
2. Nurses on maternity leave, sick leave, or going out to study.

Prior to the initiation of this study, ethical approval (Reference No: K202511-16) was obtained from the Medical Ethics Committee of Tangdu Hospital, Air Force Medical University. The clinical investigation was conducted after submitting a written application and receiving approval. According to the sample size requirements (19), the minimum sample size was 10–20 times the number of independent variables. The independent variables used in this study were 18 (12 items of general information + 3 dimensions of moral resilience scale + 3 dimensions of ethical behavior scale). Considering a 10% sample dropout rate and invalid questionnaires, the minimum sample size was *n* = 18 × 10 = 180. The minimum number of enrolled patients was 180÷(1–10%) = 200, and the actual number of enrolled patients in this study was 386.

### Study design: a cross-sectional survey study

#### Survey tools

General information questionnaire: self—made, including gender, age, marital status, education level, living style, hospital level, department, monthly income, number of monthly night shifts, nursing years, whether one is a specialist nurse, and whether one has participated in ethics—related knowledge training/meetings, etc.

The Chinese version of the Rushton moral resilience Scale (RMRS) was compiled by Rushton in 2021 (20). It was translated into Chinese by Chen (21), a Chinese scholar, and its reliability and validity were tested among nurses in 2024.

The scale consists of three dimensions and 17 items in total, including coping with nursing ethical dilemmas and relationship integrity (10 items), personal integrity (2 items), and nursing ethical efficacy (5 items). Each item is scored using a Likert 4—point scale, ranging from “strongly disagree” to “strongly agree,” with scores assigned from 1 to 4 in order. Items 2, 4, 5, 6, 8, 10, 11, 13, 14, 15, and 16 are reverse—scored. The total score of the scale ranges from 17 to 68. The higher the score, the better the moral resilience. In this study, the Cronbach’s *α* coefficient of the scale was 0.796.

Ethical Behavior Scale for Nurses-Revised (EBSN-R), which was translated into Chinese by Chen Qihui et al. in 2023. The EBSN-R includes 3 dimensions: quality care, equitable care, and risk prevention, with 5 items each. Each item is scored on a 6-point scale, ranging from 1 to 6 points, from “very inconsistent” to “very consistent.” The total score ranges from 15 to 90 points. The higher the score, the better the nurses’ ethical behavior practice ability in the clinical environment. The Cronbach’s *α* coefficient of the scale is 0.892, and the Cronbach’s α coefficient of the scale in this study is 0.880.

#### Data collection methods

Electronic questionnaires were generated using the Wenjuanxing platform (Changsha Ranxing Information Technology Co., Ltd., Changsha, China). The survey link (via QR code) was distributed through a hierarchical cascade involving hospital nursing directors, ward head nurses, and clinical nurses to ensure that respondents were licensed clinical registered nurses. Clear instructions are provided on the homepage and each section of the questionnaire. The same IP address can only be used to fill out the questionnaire once within the same time frame. The questionnaire survey was conducted anonymously, without disclosing personal information or separately discussing personal factors.

For the test—retest reliability method of the questionnaire, three different questions with the same correct order of choices were placed in different positions. Questionnaires with inconsistent answers for these three questions were eliminated. Questionnaires with response times shorter than 5 min, or those where the same option was chosen for more than 10 questions, were regarded as invalid. This was done to ensure the reliability of the questionnaire and to guarantee that each questionnaire could genuinely reflect the true intentions of clinical nurses.

Initially, a total of 386 questionnaires were distributed, and 338 valid questionnaires were returned, with an effective recovery rate of 87.56%. The differences between the two input results were identified through automatic comparison, and a third—party re—checked and corrected the data to finally form a complete and accurate data set. Simultaneously, numerical tests and time analysis were employed to further exclude abnormal data.

### Statistical methods

SPSS 28.0 and Mplus 8.3 statistical packages were used for data statistics and analysis. Measurement data were described by the mean and standard deviation, and enumeration data were described by frequency and percentage (%). In this study, the *t*-test and chi-square test were used to analyze the influencing factors between groups to evaluate the influence of various factors on different latent profiles, and the significance level was set at *p* < 0.05. Mplus 8.3 software was used to conduct latent profile category analysis of the moral resilience of clinical nurses. Firstly, the data model C1 was established, and the number of categories was gradually increased. The best—fitting model was screened by comparing the log—likelihood function value [Log(L)], Akaike information criterion (AIC), Bayesian information criterion (BIC), and the sample—corrected BIC (aBIC) value. In addition, the Ro—Mondell—Reuben corrected likelihood ratio (LRT), bootstrap—based likelihood ratio test (BLRT), and *p* value were commonly used to evaluate the degree of model fit. The entropy value is used to evaluate the accuracy of classification. The closer the value is to 1, the higher the accuracy of classification is.

## Results

### Basic information and moral resilience scale scores

A total of 386 questionnaires were distributed, and 48 invalid questionnaires were excluded, with an effective recovery rate of 87.56%. The information of clinical nurses is shown in Table 1.

**Table 1:** General information of the respondents (*n* = 338).

Items	Categories	*n*	Percentage (%)
Gender	Male	42	12.43
	Female	296	87.57
Age (years)	<30	131	38.76
	30 ~ 39	122	36.09
	40 ~ 49	63	18.64
	≥50	22	6.51
Marital status	Unmarried	108	31.95
	Married	210	62.13
	Divorce	20	5.92
Level of education	Technical secondary school or below	18	5.33
	Junior college	120	35.50
	Undergraduate	174	51.48
	Graduate student	26	7.69
Place of residence	Town	338	100.00
	Rural	0	–
Nursing age (years)	< 5	78	23.08
	5 ~ 9	126	37.28
	10–19	86	25.44
	≥20	48	14.20
Style of residence	Rental	89	26.33
	Purchase	230	68.05
	Others	19	5.62
Hospital grade	Second grade	29	8.58
	Triple B	89	26.33
	Third grade	220	65.09
Department	Internal medicine	66	19.53
	Surgery	65	19.23
	Outpatient and emergency department	76	22.49
	Obstetrics and gynecology	26	7.69
	ICU	87	25.74
	Other	18	5.33
Monthly income (Yuan)	<5,000	32	9.47
	5,000 -	91	26.92
	10,000 -	215	63.61
Number of monthly night shifts	<3	63	18.64
	3 ~ 6	188	55.62
	≥7	87	25.74
Specialist nurse	Is	182	53.85
	No	156	46.15
Participate in ethics training	Is	198	58.58
	No	140	41.42

### Common method bias

Common Method Bias (CMB) was assessed using Harman’s single-factor test. The results revealed that 10 factors possessed eigenvalues greater than 1, with the first factor explaining 22.05% of the variance, which fell below the critical threshold of 40%. This indicates that common method bias was not a significant concern in this study.

### Moral resilience and ethical behavior scores of clinical nurses

Clinical nurses scored (44.27 ± 5.33) on moral resilience and (41.08 ± 6.34) on ethical behavior. Detailed scores for each dimension and item are presented in Table 2.

**Table 2:** Moral resilience and ethical behavior scores of clinical nurses.

Items	Total score	Item mean score
Total moral resilience score	44.27 ± 5.33	2.60 ± 0.45
Coping and relationship integrity dimensions	26.23 ± 4.18	2.62 ± 0.56
Personal integrity dimension	5.29 ± 1.24	2.64 ± 0.44
Nursing ethical efficacy dimension	12.75 ± 2.64	2.55 ± 0.37
Total score of ethical behavior	41.08 ± 6.34	2.74 ± 0.53
Quality care dimension	13.91 ± 2.53	2.78 ± 0.49
Equitable nursing dimension	14.03 ± 2.49	2.81 ± 0.58
Risk prevention dimension	13.14 ± 2.71	2.63 ± 0.46

Theoretical score range: RMRS = 17–68; EBSN-R = 15–90.

### Latent profile analysis of moral resilience of clinical nurses

In this study, beginning with a single—category initial model, one category is added successively. As the number of profile categories increases, more fitting indicators emerge: When LRT and BLRT are significant, the smaller the AIC, BIC, and aBIC values are and the larger the Entropy value is, the higher the degree of fit. The three-profile solution demonstrated superior fit over the two- and four-profile models across all evaluated indices, supporting the identification of three distinct subgroups (Table 3).

**Table 3:** Comparison of fitting parameter indexes of different latent profile models (*n* = 338).

Models	AIC	BIC	aBIC	Entropy	LRT	BLRT	Class probability
1	26753.257	26784.215	26764.827				
2	26027.914	26127.253	26031.903	0.942	0.000	<0.001	0.31/0.69
3	25136.986	25339.714	25238.820	0.972	0.006	<0.001	0.28/0.46/0.26
4	25041.537	25256.818	25154.838	0.990	0.112	<0.001	0.23/0.27/0.18/0.32

### Average attribution rate of three latent categories of moral resilience of clinical nurses

To verify the reliability of the three latent profile category analysis results, the average attribution probability of each of the three class samples was calculated. The results show that the average attribution probability ranges from 96.8 to 98.0%, all of which are greater than 90%, indicating that the three latent profiles are reliable, as shown in Table 4.

**Table 4:** Average attribution rate of three types of moral resilience (*n* = 338).

Model	C1	C2	C3
C1	0.968	0.020	0.012
C2	0.016	0.976	0.008
C3	0.016	0.004	0.980

### Latent category characteristics of moral resilience of clinical nurses

**Figure 1:**
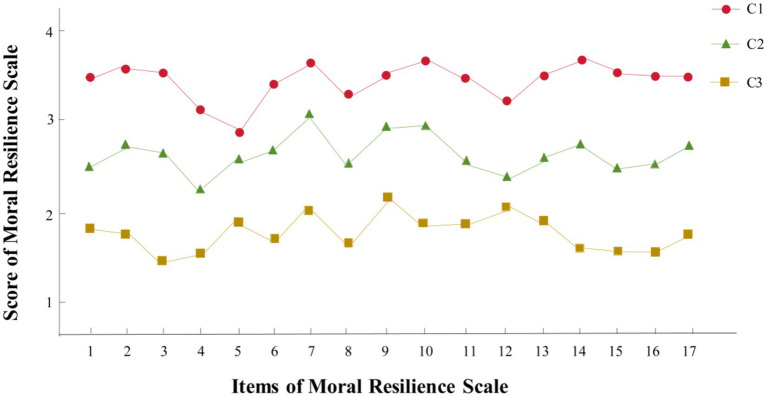
Line chart of latent category characteristics of moral resilience of clinical nurses.

According to the results of latent profile analysis, we found that clinical nurses can be divided into three categories. By creating line plots of the scores of each dimension of the Moral Resilience Scale (see Figure 1), we can more clearly analyze the characteristics of moral resilience of clinical nurses in these three latent categories. The three latent categories were named as follows: Category 1 (C1) was the “high moral resilience group,” accounting for 28.1% (95 cases); Category 2 (C2) was the “medium moral resilience group,” accounting for 46.2% (156 cases); and Category 3 (C3) was the “low moral resilience group,” accounting for 25.7% (87 cases).

### Univariate analysis of latent categories of moral resilience of clinical nurses

Univariate analysis showed that there were significant differences in nursing age (χ^2^ = 12.916, *p* = 0.044), hospital grade (χ^2^ = 14.006, *p* = 0.007), whether they were specialized nurses (χ^2^ = 16.721, *p* < 0.001), and whether to participate in ethical training (χ^2^ = 14.237, *p* < 0.001) among the three categories of nurses, as shown in Table 5.

**Table 5:** Univariate analysis of latent profiles of different moral resilience (*n* = 338).

Variables	Classification	Group C1 (*n* = 95)	Group C2 (*n* = 156)	Group C3 (*n* = 87)	χ^2^	*p*-value
Gender	Male	11	24	7	2.851	0.240
	Female	84	132	80		
Age (years)	<30	34	61	36	1.427	0.964
	30 ~ 39	35	59	28		
	40 ~ 49	19	27	17		
	≥50	7	9	6		
Marital status	Unmarried	23	51	34	5.494	0.240
	Married	67	94	49		
	Divorced	5	11	4		
Level of education	Technical secondary school or below	5	7	6	2.681	0.848
	Junior college	35	58	27		
	Undergraduate	46	79	49		
	Graduate student	9	12	5		
Nursing age (years)	<5	18	33	27	12.916	0.044
	5 ~ 9	27	64	35		
	10–19	32	38	16		
	≥20	18	21	9		
Style of residence	Rental	24	44	21	0.615	0.961
	Purchase	66	103	61		
	Others	5	9	5		
Hospital grade	Second grade	6	9	14	14.006	0.007
	Third B	18	44	27		
	Third class A	71	103	46		
Department you work in	Internal medicine	15	34	17	9.307	0.503
	Surgery	16	31	18		
	Outpatient and emergency departments	21	38	17		
	Obstetrics and gynecology	5	11	10		
	ICU	33	35	19		
	Others	5	7	6		
Monthly income (yuan)	<5,000	8	15	9	0.440	0.979
	5,000 -	27	40	24		
	10,000 -	60	101	54		
Number of night shifts per month	<3	12	31	20	4.428	0.351
	3 ~ 6	55	84	49		
	≥7	28	41	18		
Specialist nurse	Yes	67	79	36	16.721	< 0.001
	No	28	77	51		
Participate in ethics training	Yes	71	81	46	14.237	0.001
	No	24	75	41		

### Logistic regression analysis of latent categories of moral resilience of clinical nurses

Logistic regression analysis was conducted with the moral resilience grouping of clinical nurses as the dependent variable and the indicators with statistically significant differences in univariate analysis as the independent variables. The results showed that with group C3 as the reference, the assignment values were as follows: nursing age (< 5 years = 1, 5–9 years = 2, 10–19 years = 3, ≥20 years = 4), hospital level (second - class A = 1, third - class B = 2, third - class A = 3), whether being specialized nurses (no = 0, yes = 1), and whether to participate in ethics training (no = 0, yes = 1). Logistic regression analysis showed that nursing age, hospital level, being a specialist nurse, and ethics training were the influencing factors of moral resilience (*p* < 0.05 see Table 6).

**Table 6:** Logistic regression analysis of the change trajectory of moral resilience among clinical nurses (*n* = 338).

Items	*B*	*SD*	Walds χ^2^	*P*-value	OR	95% CI
C1 vs. C3						
Constants	1.679	0.357	22.119	<0.001	–	–
Nursing age (years)						
5 ~ 9	0.213	0.094	5.135	0.031	1.237	1.029 ~ 1.488
10–19	0.370	0.125	8.762	0.003	1.448	1.133 ~ 1.850
≥20	0.468	0.168	7.760	0.010	1.597	1.149 ~ 2.219
Hospital grade						
Grade 3 B	0.304	0.141	4.648	0.038	1.355	1.028 ~ 1.787
Third class A	0.401	0.154	6.780	0.019	1.493	1.104 ~ 2.019
Specialist nurse						
Yes	0.542	0.199	7.418	0.012	1.719	1.164 ~ 2.540
Attend ethics training						
Yes	0.627	0.205	9.355	<0.001	1.872	1.253 ~ 2.798
C2 vs. C3						
Constants	1.416	0.312	20.598	<0.001	–	–
Nursing age (years)						
5 ~ 9	0.194	0.085	5.209	0.030	1.214	1.028 ~ 1.434
10–19	0.332	0.118	7.916	0.009	1.394	1.106 ~ 1.756
≥20	0.395	0.131	9.092	0.001	1.484	1.148 ~ 1.919
Hospital grade						
Grade 3 B	0.256	0.127	4.063	0.046	1.292	1.007 ~ 1.657
Third Class A	0.346	0.149	5.392	0.029	1.413	1.055 ~ 1.893
Specialist nurse						
Yes	0.477	0.163	8.564	0.005	1.611	1.171 ~ 2.218
Attend ethics training						
Yes	0.524	0.194	7.296	0.014	1.689	1.155 ~ 2.470

### Analysis of differences in ethical behavior of clinical nurses with different profiles of moral resilience

The level of ethical behavior of clinical nurses in group C1 was significantly higher than that in group C2 (*p* < 0.01) and significantly higher than that in group C3 (*p* < 0.01). Moreover, the level of ethical behavior of clinical nurses in group C2 was significantly higher than that in group C3 (*p* < 0.01 see Table 7 for details).

**Table 7:** Differences analysis of ethical behavior of clinical nurses with different moral resilience profiles.

Variables	Group C1	Group C2	Group C3	*F*	*P*	*η^2^*	Compare the results in pairs
Quality care dimensions	16.19 ± 2.95	14.12 ± 2.41	12.05 ± 2.78	23.567	<0.001	0.124	C1 > C3^**^, C1 > C2^**^, C2 > C3
Dimensions of equitable care	15.52 ± 2.30	13.94 ± 2.65	11.96 ± 2.73	19.164	<0.001	0.104	C1 > C3^**^, C1 > C2^**^, C2 > C3
Risk prevention dimension	13.92 ± 2.21	13.08 ± 2.75	11.99 ± 2.67	5.648	0.012	0.033	C1 > C3^**^, C2 > C3
Total ethical behavior score	45.63 ± 7.01	41.14 ± 6.29	36.00 ± 5.59	117.349	<0.001	0.413	C1 > C3^**^, C1 > C2^**^, C2 > C3

*indicates *P* < 0.05, **indicates *P* < 0.01.

## Discussion

### The moral resilience of clinical nurses is at a medium level

The results of this study indicate that the moral resilience of clinical nurses is at a moderate level (item mean score: 2.60 ± 0.45). This finding is generally consistent with previous domestic studies (11), suggesting that while Chinese clinical nurses possess a certain foundation of psychological resilience when facing ethical dilemmas, there remains considerable room for improvement. This moderate status reflects both the resilience accumulated by the nursing workforce through long-term practice and the systemic challenges they currently face (22, 23). These challenges are primarily attributed to the uneven distribution of medical resources in China, the “high-load, high-risk” working conditions driven by a large patient population, and the relative insufficiency of institutional ethical support systems (e.g., accessibility of ethics committees, routine ethical case supervision) and systematic contextualized ethics education (24, 25). Therefore, transitioning from a “moderate” to an “advanced” level requires urgent systemic interventions from hospital administrators at the organizational level, including optimizing workloads, establishing institutionalized ethical support networks, and deepening ethics education.

### There were three different profile categories of moral resilience among clinical nurses

This study, through latent profile analysis, identified three heterogeneous categories of moral resilience among clinical nurses: the high-resilience group, the moderate-resilience group, and the low-resilience group. This classification holds significant theoretical and practical implications. Theoretically, it challenges the simplistic assumption that nurses’ moral resilience exists as a single continuum, confirming instead the presence of significant, qualitative differences within the group (17, 26). This suggests that the formation of moral resilience is not influenced by a single linear factor but rather by the complex interplay of individual traits, environmental resources, and accumulated experience. In practice, this classification provides a direct basis for implementing precise, stratified management interventions. Nurses in the high-resilience group typically possess greater psychological resilience, positive coping strategies, and strong social support, enabling them to serve as “ethical backbones” within their departments (27, 28). The moderate-resilience group, which constitutes the majority, is more susceptible to environmental fluctuations in their resilience and represents the key target for intervention and improvement. The low-resilience group, however, is at high risk for ethical fatigue and job burnout, necessitating prioritized attention and support (29, 30). Differentiating among these three groups implies that nursing management strategies should shift from “universal” training to “personalized” support, thereby achieving optimal resource allocation and maximizing intervention effectiveness.

### Analysis of influencing factors of three different profile categories of moral resilience among clinical nurses

Previous studies have indicated a positive correlation between age, length of service, and moral resilience, as senior nurses, who have accumulated extensive clinical experience and coping strategies, typically exhibit higher levels of moral resilience compared to their junior counterparts. Furthermore, nurses who are married, possess higher educational qualifications, and have undergone systematic ethics training tend to achieve relatively higher scores in moral resilience (31, 32). In addition, the hospital ethical climate has been confirmed as the strongest predictor. The results of this study indicate that years of nursing experience, hospital level, whether one is a specialized nurse, and participation in ethics training are influential factors of moral resilience. This is highly consistent with the findings of Zheng et al. and Li et al. (33, 34), both of which emphasize the core roles of work experience (e.g., nursing age), organizational environment (e.g., hospital grade), and ethical education in shaping moral resilience. Nurses who participate in ethics training generally perceive a more positive hospital ethical climate, which aligns broadly with the findings of this study. These factors are deeply embedded within the characteristics of China’s healthcare system. As years of nursing experience increase, nurses continuously accumulate experience in handling ethical dilemmas through clinical practice, enabling them to more accurately identify ethical issues and more effectively apply ethical decision-making models. Consequently, they are better able to maintain stronger psychological resilience and coping capabilities when facing moral conflicts. The positive influence of years of nursing experience confirms the “experience empowerment” pathway, as clinical nurses in China gradually accumulate “practical wisdom” in dealing with localized ethical challenges while managing a high volume of complex cases and navigating interpersonal interactions within the doctor-patient dynamic in a relationship-oriented society (35). The impact of hospital level on moral resilience reflects the shaping role of the organizational environment on individual ethical development. In China, higher-level hospitals, such as tertiary grade A hospitals, generally have more comprehensive ethics committee structures, continuing education resources, and multidisciplinary collaboration models. These hospitals are better equipped to provide nurses with systematic ethics training and ongoing ethical guidance, and such organizational-level support significantly enhances nurses’ moral sensitivity and decision-making capabilities (36, 37). The positive predictive effect of being a specialized nurse on moral resilience stems from the specialized training and in-depth practice that these nurses receive in specific fields. This equips them with stronger professional confidence and ethical judgment when confronting ethical challenges unique to their specialties (38, 39). Participation in ethics training, as an intervenable factor, operates by systematically imparting ethical knowledge, conducting case analyses, and simulating scenarios. This approach helps nurses master the fundamental principles and methods of ethical decision-making, enhances their ethical sensitivity and moral courage, and thereby enables them to make ethically sound decisions and maintain psychological balance when facing ethical dilemmas (34, 40). However, current domestic training often focuses heavily on theoretical instruction. In the future, it is necessary to strengthen reflective, scenario-based training driven by real clinical cases from China to more effectively enhance nurses’ coping abilities. Together, these influencing factors constitute a multidimensional support system for nurses’ moral resilience. This suggests that nursing managers should develop comprehensive intervention strategies at the individual, organizational, and educational levels. By implementing stratified training, strengthening organizational support systems, and reinforcing ethics education, the moral resilience level of clinical nurses can be comprehensively improved, thereby promoting the continuous enhancement of nursing quality.

### The correlation between different levels of moral resilience and ethical behavior of clinical nurses and its guiding effect on clinical practice

This study found that the ethical behavior level of clinical nurses is moderate. Furthermore, the ethical behavior level of nurses in the high moral resilience group was significantly higher than that of those in the medium and low moral resilience groups, and the ethical behavior level of the medium resilience group was significantly higher than that of the low resilience group. This finding aligns with conclusions from international research and has been validated within the Chinese context (41). It clearly reveals that moral resilience serves as the internal psychological engine driving ethical behavior. Nurses with high resilience, due to their stronger emotional regulation and cognitive restructuring abilities, are more likely to adhere to professional standards and take constructive actions when facing ethical dilemmas characteristic of the Chinese context, such as “family members concealing a patient’s condition” (42). In contrast, nurses with low resilience, often due to emotional exhaustion and a sense of powerlessness, may exhibit avoidance or rigid compliance. This correlation has direct implications for clinical management: improving the ethical behavior level of nursing teams cannot rely solely on normative indoctrination; it is essential to start by cultivating their internal moral resilience. By fostering a supportive ethical atmosphere, establishing mechanisms for sharing and supervising “ethical dilemma” cases, and identifying and supporting nurses with low resilience, psychological resources can be effectively translated into clinically ethical behaviors. This, in turn, ultimately safeguards patient safety and enhances the quality of nursing care (43, 44).

The three hypotheses (H1-H3) proposed in this study were all supported by the data. First, latent profile analysis identified three distinct moral resilience profiles with statistical significance, confirming the existence of high-resilience, moderate-resilience, and low-resilience subgroups (supporting H1). This demonstrates significant heterogeneity in the intrinsic psychological resources of the nursing population when coping with ethical dilemmas. Second, systematic differences were observed across profiles in key variables: nurses in the high-resilience group had longer nursing experience and perceived a significantly more positive organizational ethical climate (supporting H2), suggesting that professional experience and specialized practice environments are important contextual factors shaping high-level moral resilience. Finally, profile classification significantly predicted the level of ethical behavior: the high-resilience group scored highest in ethical behavior, followed by the moderate-resilience group, while the low-resilience group scored lowest (supporting H3). It is particularly noteworthy that the results of the ANOVA revealed a substantial effect size for differences in total ethical behavior scores among the different moral resilience profiles (partial η^2^ = 0.413). This large effect size indicates that the disparities in moral behavior are not only statistically significant (*F* = 117.349, *p* < 0.001) but also possess substantive clinical significance; specifically, the moral resilience profile accounts for 41.3% of the variance in ethical behavior, representing a considerable difference that cannot be overlooked in nursing management practice. These findings not only validate the core role of moral resilience as a key psychological resource for ethical behavior but also reveal, from a subgroup perspective, that nurses’ moral resilience can be maximally translated into stable, high-level ethical behavior only when cognitive and emotional dimensions are balanced and when sufficient organizational resources and support are available. This provides direct empirical evidence for future targeted and stratified intervention strategies.

## Limitations and future directions

This study has several limitations. First, regarding sampling methods, the use of convenience sampling may have resulted in insufficient sample representativeness, limiting the generalizability of the findings. Second, regarding measurement methods, although self-report scales were used and common method bias testing was conducted, it remains difficult to completely eliminate the potential impact of social desirability bias on result accuracy. Third, regarding research design, the cross-sectional survey design only captures data at a single time point, making it impossible to determine causal relationships and limiting causal inference. Fourth, regarding measurement consistency, which must be explicitly pointed out: this study did not conduct Measurement Invariance Testing across different hospital levels (e.g., tertiary vs. secondary hospitals). If systematic differences exist in the factor structure or thresholds of the moral resilience scale across various healthcare settings, the statistical validity of cross-group comparisons (such as regression analyses examining the influence of hospital level) may be compromised. Finally, regarding variable inclusion, the study focused primarily on individual-level variables while overlooking critical organizational environmental factors, such as hospital ethical climate and leadership support, which may play important moderating roles in the formation and mechanisms of moral resilience.

Future research should integrate individual and contextual factors (e.g., ethical climate), exploring the buffering effects of organizational resources based on Conservation of Resources Theory. Additionally, tailored, stratified intervention programs targeting low-resilience groups (e.g., mindfulness training, ethical simulations) should be developed and validated via randomized controlled trials to construct systematic models and enhance the ethical practice competence of nursing teams.

## Summary

In this study, latent profile analysis was employed to classify the moral resilience of clinical nurses into three latent profile categories, namely high-, medium-, and low-level groups. These three latent profile categories were influenced by nursing experience, hospital level, whether they were specialist nurses, and participation in ethical training. The moral resilience of different profiles is closely associated with the level of ethical behavior of clinical nurses. These findings directly inform specific clinical management pathways: nursing managers should abandon “one-size-fits-all” approaches and implement stratified interventions based on the heterogeneous characteristics of moral resilience—specifically, establishing early warning and psychological support mechanisms for the low-resilience group, reinforcing ethical decision-making training for the moderate-resilience group, and integrating high-resilience nurses into departmental ethical supervision systems. Additionally, given the protective effects of nursing age, specialist identity, and ethics training, it is recommended to construct a collaborative, whole-process ethics education system linking academic institutions and hospitals, with a focus on increasing ethical resource investment in secondary and primary healthcare institutions. Future research should expand sample representativeness and conduct multicenter longitudinal studies to elucidate the dynamic evolution of moral resilience across professional cycles and to develop precise intervention programs tailored to the local context.

## Data Availability

The original contributions presented in the study are included in the article/supplementary material, further inquiries can be directed to the corresponding author.
