# Correction: Latent profile analysis of moral resilience among clinical nurses and its association with ethical behavior: a multi-hospital cross-sectional study in China

**DOI:** 10.3389/fpubh.2026.1928111

**Published:** 2026-07-23

**Authors:** Xiaohan Shi, Ying Zhang, Hongjuan Lang, Rui Wang, Yang Wang, Aiping Sun, Yuan Hui

**Affiliations:** 1 Healthcare Center for Military Personnel, Tangdu Hospital, The Fourth Military Medical University, Xi'an, Shaanxi, China; 2 Department of Nursing, The Fourth Military Medical University, Xi'an, Shaanxi, China; 3 Department of Ophthalmology, Tangdu Hospital, The Fourth Military Medical University, Xi'an, Shaanxi, China

**Keywords:** clinical nurses, ethical behavior, influencing factors, latent profile analysis, moral resilience, relevance

Affiliation [Department of Ophthalmology, Tangdu Hospital, The Fourth Military Medical University, Xi'an, Shaanxi, China] was erroneously given as [Department of Ophthalmology, The Second Affiliated Hospital of The Fourth Military Medical University, Xi'an, Shaanxi, China].

The original version of this article has been updated.

